# Fatty Acid Composition and Antioxidant Activity of Tea (*Camellia sinensis* L.) Seed Oil Extracted by Optimized Supercritical Carbon Dioxide

**DOI:** 10.3390/ijms12117708

**Published:** 2011-11-08

**Authors:** Yuefei Wang, Da Sun, Hao Chen, Lisheng Qian, Ping Xu

**Affiliations:** Department of Tea Science, Zhejiang University, Hangzhou 310058, China; E-Mails: tyfwang@gmail.com (Y.W.); 20916167@zju.edu.cn (D.S.); qcws2003@126.com (H.C.); tlshqian@gamil.com (L.Q.)

**Keywords:** tea, seed oil, supercritical carbon dioxide (SC-CO_2_), response surface methodology (RSM), antioxidant activity

## Abstract

Seeds are another product in addition to leaves (raw materials for teas) of tea (*Camellia sinensis* L.) plant. The great increase of tea consumption in recent years raises the challenge of finding commercial applications for tea seeds. In the present study, supercritical carbon dioxide (SC-CO_2_) extraction edible oil from tea seed was carried out, response surface methodology (RSM) was used to optimize processing parameters including time (20–90 min), temperature (35–45 °C) and pressure (50–90 MPa). The fatty acid composition and antioxidant activity of the extracted oil was also investigated. The highest yield of oil (29.2 ± 0.6%) was obtained under optimal SC-CO_2_ extraction conditions (45 °C, 89.7 min and 32 MPa, respectively), which was significantly higher (*p* < 0.05) than that (25.3 ± 1.0%) given by Soxhlet extraction. Meanwhile, tea seed oil extracted by SC-CO_2_ contained approximately 80% unsaturated fatty acids and showed a much stronger scavenging ability on the DPPH radical than that extracted by Soxhlet. SC-CO_2_ is a promising alternative for efficient extraction of edible oil from tea seed. Moreover, tea seed oil extracted by SC-CO_2_ is highly edible and has good antioxidant activity, and therefore may play a potential role as a health-promoting food resource in human diets.

## 1. Introduction

Seeds are another product in addition to leaves (raw materials for green tea, black tea, oolong tea, *etc*.) of tea (*Camellia sinensis* L.) plant. With an annual production of over three million tons [1], tea is the second most popular beverage in the world. As tea has become more and more popular recently, the yield of tea seed has also increased. For example, in China, over a million tons of tea seed are produced each year [2]. The great increase in amount of tea seed raises the challenge of finding suitable commercial applications. Like other *Camellia* genera from the Theaceae family, tea seeds are rich in oil (30–32%) [3], which is considered a kind of edible oil with high quality because the predominant fatty acids (FAs) are the monounsaturated fatty acid (MUFA) oleic acid and the polyunsaturated fatty acid (PUFA) linoleic acid [4]. Oleic acid is essential in human nutrition and helps to reduce levels of LDL-cholesterol, total cholesterol and the glycemic index [5]. Linoleic acid, as an essential fatty acid from omega-3 group, is important in the development and maintenance of the nervous system and physiological functions in humans [6]. Besides, tea seed oil was shown to have an anti-obesity effect in an *in vivo* model [7]. Thus, using tea seeds as a source of edible oil has been suggested as a solution to utilizing what would otherwise be a waste product.

Generally, the conventional industrial methods used to produce seed oil are organic solvent extraction (mainly using petroleum ether, petroleum benzene and hexane) and mechanical pressing. However, since solvent extraction causes environmental and safety issues and mechanical pressing gives only a low yield, a safer and more efficient extraction technique is required for tea seed oil production. Supercritical carbon dioxide (SC-CO_2_) extraction has attracted increasing attention recently due to its advantages of being nontoxic, nonflammable, inexpensive and producing a high yield of oil. Previously, Rajaei *et al*. [8] tried to use SC-CO_2_ with a modifier of ethanol to recover oil from tea seed as well. Despite ethanol being able to enhance oil yield and extract some minor antioxidants with relatively high polarity, it is supposed to be unsuitable for extraction of edible oils because of the high temperature required for recovery of the ethanol which may reduce the edible quality of the oil, and residual ethanol in the oil is also a source of concern. Therefore, using CO_2_ as the only solvent is preferred and has been successfully employed in extraction of oils from a wide range of seeds, including rosehip [9], flax [10], sunflower and rape [11], pumpkin [12] and chia [13].

Response surface methodology (RSM) is used to evaluate the effects of multiple factors and their interactions on one or more response variables, and is therefore suitable to find the optimum combination of factor levels [14]. Recently, RSM has been successfully employed to optimize SC-CO_2_ extraction of oils from a variety of materials [15–18].

The objectives of this study were to employ SC-CO_2_ to extract oil from tea seed, to use RSM to determine the optimal conditions, including pressure, temperature and dynamic time, to determinate the fatty acid composition of the extracted oil using gas chromatographic (GC), and to evaluate the antioxidant activity of the extracted oil by 2,2-diiphenly-1-picrylhydrazyl (DPPH) free radical scavenging assay.

## 2. Results and Discussion

### 2.1. Model Fitting

Experiment sets and the corresponding oil yield obtained are given in Table 1. The ANOVA analysis of the quadratic regression models for oil yield showed that the model was significant (*p* < 0.01) with an F-value of 89.63 (Table 2). The *p*-value of the “lack of fit” of the model was 0.6932, which implied that the “lack of fit” was not significant relative to the error [19]. The coefficient of determination (*R*^2^) and adjusted coefficient of determination (Adj. *R*^2^) were 0.9878 and 0.9767, respectively, which indicated that the accuracy of the polynomial model was adequate. The second-order polynomial model was expressed by the following quadratic equation:

$$Y=27.23-0.014X_{1}+3.45X_{2}+2.86X_{3}-025{X_{1}}^{2}-1.66{X_{2}}^{2}-1.38{X_{3}}^{2}+0.59X_{1}X_{2}+0.19X_{1}X_{3}-2.06X_{2}X_{3}$$

where *Y* represents the oil yield, *X*_1,_ *X*_2_ and *X*_3_ are the levels of temperature, time and pressure, respectively. It was observed that the factor with the largest effect on oil yield was linear extraction time (*p* < 0.01), followed by extraction pressure (*p* < 0.01), and the interaction between time and pressure (*p* < 0.01). The interaction between temperature and time also had a significant effect on oil yield (*p* < 0.05), whereas, the linear extraction temperature and the interaction of temperature and pressure showed no statistically significant (*p* > 0.05) impact on oil yield. All of the second-order terms of extraction parameters had negative effects on oil yield.

### 2.2. Response Surface Analysis

**Figure 1:**
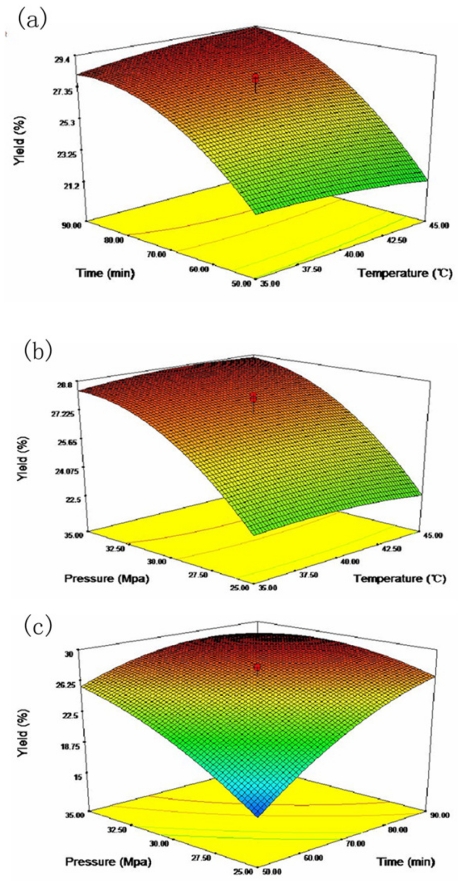
Response surfaces plots of SC-CO_2_ extraction of tea seed oil. Yield of oil is presented as a function of: (**a**) time and temperature (extraction pressure set at 30 MPa); (**b**) pressure and temperature (extraction time set to 70 min); and (**c**) pressure and time (extraction temperature set to 40 °C).

Response surfaces can be illustrated on three-dimensional plots by presenting the response as a function of two factors and keeping the other constant. The influence of extraction time and temperature on oil yield at a fixed pressure of 30 MPa is shown in (Figure 1a). Yield decreased slightly with increasing extraction temperature when extraction time was relatively shorter (around 50 min), but increased with longer extraction time. The effect of extraction pressure and temperature on oil yield at a fixed extraction time of 70 min is presented in (Figure 1b). It can be found that temperature showed a slight effect on oil yield regardless of extraction pressure, the influence of pressure on oil yield at a fixed temperature was observed to be similar with that of time (Figure 1a). The influence of pressure and time at a temperature of 40 °C is shown in (Figure 1c). A linear increase in yield with the increase of pressure at low level of time, while an obvious quadratic effect on yield of oil was observed when extraction time was longer (around 90 min). The effect of time on oil yield at fixed pressures was similar to that of pressure.

It is well-known that temperature has a dual effect on SC-CO_2_ extraction. Generally, higher temperature could accelerate mass transfer and improve the extraction yield [20]. However, increasing temperature would reduce the solvent density and decrease the yield at pressures in the critical range [21]. Consequently, solubility of the solute at a constant pressure was likely to depend on whether solvent density or solute vapor pressure was higher [22]. In our study, extraction temperature showed a minor effect on yield, an almost constant oil yield was observed with increasing extraction temperature (Figure 1). A similar phenomenon was also observed in SC-CO_2_ extraction of chia seed oil [13]. That may be explained by the fact that a dynamic balance was maintained between solvent density and vapor pressure under the experimental conditions. Extraction time and pressure were found to be main parameters that influenced yield of tea seed oil by SC-CO_2_ extraction in our study. Increasing pressure caused an increase in supercritical CO_2_ density, resulting in an enhanced solubility of solutes. However, high pressure is not preferred due to increased repulsing solute-solvent interactions resulting from highly compressed CO_2_ at high pressure, which potentially induced complex extraction and difficult analysis. Despite four parameters, including miscibility and threshold, the pressure at which the solute reaches its maximum solubility, the fractionation pressure range, and a knowledge of the physical properties of the solute, were suggested to be critical to understand the solute behavior in supercritical media; the underlying mechanisms of various performance of SC-CO_2_ extraction under different conditions have not been clearly illustrated yet.

Based on the above model, the optimal values for temperature, time and pressure were predicted to be 45 °C, 89.7 min and 32 MPa, respectively, to give a maximum yield of 29.5%. Validation experiments carried out under these optimal conditions found the yield to be 29.2 ± 0.6%, which was not significantly different from the predicted value (*p* > 0.05). These results indicated that the quadratic model was reliable.

### 2.3. Comparison of SC-CO_2_ Extraction with SE

#### 2.3.1. Oil Yields

The total yield of tea seed oil by Soxhlet extraction was 25.3 ± 1.0%, significantly (*p* < 0.05) lower than that (29.2 ± 0.6%) by using SC-CO_2_ under optimal conditions. Moreover, Soxhlet extraction needed longer time (8 h) and higher temperature (60 °C) than SC-CO_2_ extraction. Despite SC-CO_2_ extraction costing more than traditional methods, CO_2_ has higher efficiency and is non-toxic. Therefore, from a long-term view, SC-CO_2_ could be an alternative for extraction of tea seed oil.

#### 2.3.2. Fatty Acid Composition of Tea Seed Oils

**Figure 2:**
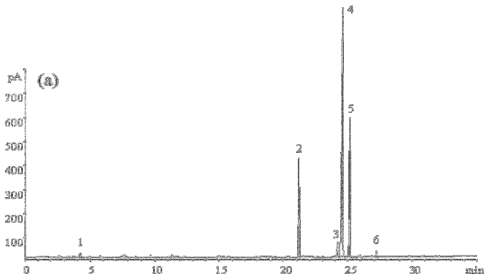

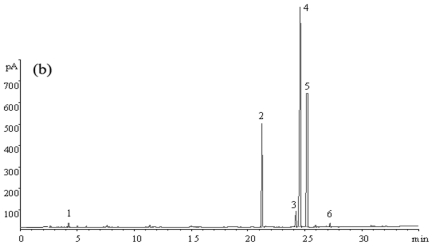
Chromatograms of fatty acid methyl esters (FAMEs) of tea seed oils. (**a**) FAMEs of the oil extracted by SC-CO_2_; (**b**) FAMEs of the oil extracted by Soxhlet. Peaks are defined as follows: 1: heptylic acid; 2: palmitic acid; 3: stearic acid; 4: oleic acid; 5: linoleic acid; and 6: gondoic acid.

The fatty acid (FA) profiles of tea seed oil extracted by SC-CO_2_ and SE were analyzed by GC (Figure 2, Table 3). Unsaturated fatty acids (UFA) make up about 80% of the total fatty acids. The most prevalent FA in tea seed oil was oleic acid, followed by linoleic acid, palmitic acid and stearic acid. This finding is consistent with previous reports on the fatty acid composition of tea seed oil cultivated in Taiwan and Japan [23], Southern India [24] and Iran [8]. The FA contents of tea seed oil obtained by SC-CO_2_ are similar to those in oil extracted by Soxhlet, although the former had a higher content of oleic acid (Table 3). Oleic acid has been found to have a cholesterol-lowering effect, among other attributes such as reducing blood pressure and the risk of stroke [25]. This study indicated that tea seed oil has a high content of UFA, and oil extracted by SC-CO_2_ contains a greater quantity of oleic acid than that extracted by Soxhlet.

#### 2.3.3. Antioxidant Activity of Tea Seed Oils

**Figure 3:**
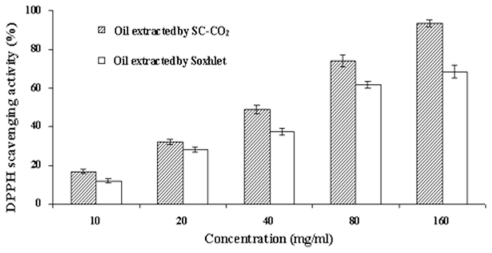
DPPH free radical scavenging activity of tea seed oils extracted by SC-CO_2_ and Soxhlet.

Samples were assayed over a range of dilutions and the results of the DPPH free radical scavenging assay are presented in Figure 3. The concentration of the sample that reduced radical absorbance by 50% (IC_50_) served as an index to compare antioxidant activity. Tea seed oil extracted using both methods showed concentration-dependent scavenging of the DPPH free radical. The yield values of tea seed oil extracted by SC-CO_2_ ranged from 17.2 ± 0.1% to 93.4 ± 1.8% when concentration of extracted oil varied from 10 to 160 mg/mL. The values for oil obtained using Soxhlet were relatively lower, ranging from 12.1 ± 2.7% to 67.8 ± 6.4% using the same range of concentrations. Oil obtained by SC-CO_2_ showed a stronger scavenging activity than that obtained by Soxhlet at all concentrations (*p* < 0.05). The IC_50_ of tea seed oil extracted by SC-CO_2_ was 35.8 mg/mL, which was nearly 40% lower than that (59.6 mg/mL) of Soxhlet-extracted oil. These results indicated that tea seed oil extracted by SC-CO_2_ had a stronger antioxidant activity than oil obtained by Soxhlet.

The antioxidant capacity and stability of tea seed oil has been previously reported [4,26]. Tea seed oil has been found to have the same antioxidant capacity as sesame oil [26] and could be used as an alternative natural antioxidant. Meanwhile, the storage stability of tea seed oil was higher than that of sunflower oil, and was similar to olive oil [4]. These properties of tea seed oil were probably due to the low content of linolenic and linoleic acid glycerides and the co-existed of antioxidants, like phytosterols, polyphenols and vitamin E [24]. In addition, kaempferol glycosides took a role in antioxidant activity of tea seed oil, since which has been found in tea seed [27]. However, these minor antioxidants may be destroyed during the long time required for Soxhlet extraction.

## 3. Experimental Section

### 3.1. Materials and Reagents

Tea seed (Jiukeng variety) was collected from the Panban tea garden (Zhejiang, China) in December 2010. Seeds were de-hulled and washed with water, then air-dried at ambient temperature. The dried seeds were milled into a powder by a pulverizer (DFY-500, Linda Machinery Co., Ltd., Zhejiang, China) and passed through a 20 mesh sieve. The resulting flour was oven-dried at 80 °C for 12 h, and then stored in a refrigerator at 4 °C until needed. Moisture content in the seed powder was 4.9% after oven-drying.

Carbon dioxide (99.99%) was obtained from Zhejiang Gas Co., Ltd. (Zhejiang, China). The Supelco fatty acid methyl ester (FAME) mix and 1,1-diphenyl-2-picrylhydrazyl (DPPH) were purchased from Sigma Aldrich (Munich, Germany). Methanol and hexane, of gas chromatography grade, were purchased from Tianjin Shield Co. Ltd. (Tianjin, China). All other chemicals and solvents were of analytical grade.

### 3.2. SC-CO_2_ Extraction

The SC-CO_2_ extraction was carried out using a supercritical fluid extractor (Spe-ed^TM^ SFE-2, Applied Separations Inc., Allentown, PA, USA). For each experiment, a 10 g sample of tea seed powder was loaded into a 50 mL thick-walled stainless steel cylindrical extractor vessel filled with defatted cotton. Liquefied CO_2_ was pumped into the extraction vessel at 2 L/min to a given pressure. The temperature inside the vessel was raised to desired ones and maintained by a heating jacket encasing the vessel. When the relevant pressure and temperature were reached, the extraction was started. Extracts were separated from the CO_2_ phase and collected. The oil samples were then weighed and yield obtained as follows:

Extraction yield (%)=(mass of extracted oil/mass of dried material)×100

### 3.3. Soxhlet Extraction (SE)

Conventional organic solvent extraction (SE) was carried out using petroleum ether (30–60 °C) to evaluate the extraction capacity and quality of oil using SC-CO_2_. A 10 g sample of milled tea seed was weighed and placed in a Soxhlet apparatus using 300 mL petroleum ether, and then continuously extracted for 8 h. After extraction, the solvent was evaporated at 60 °C under a nitrogen stream. Experiments were conducted in triplicate.

### 3.4. Experimental Design and Statistical Analysis for Response Surface Methodology

Experimental design, data analysis and quadratic model building were carried out using Design-Expert software (Trial Version 7.1.6, Stat-Ease Inc., Minneapolis, MN, USA). This software determined the optimum levels of the three independent variables of temperature (*X*_1_), time (*X*_2_) and pressure (*X*_3_) for maximum seed oil yield (*Y*). Each parameter was set to one of three levels; for temperature, the levels were 35, 40 and 45 °C; durations of extraction were 50, 70 and 90 min, and pressure levels were 25, 30 and 35 MPa (Table 4). A total of twenty experiments were performed. The experimental design was based on a central composite design (CCD) that consisted of eight (2^3^) factorial points. The six replicates for the center point were used to estimate the experimental error. All experiments were carried out in triplicate, and in a random order to minimize the effects of extraneous factors. A quadratic polynomial regression model was used to predict responses as follows:

$$Y=\beta_{0}+\sum \beta_{i}X_{i}+\sum \beta_{ii}X_{i}^{2}+\sum \sum \beta_{ij}X_{i}X_{j}$$

where *Y* represents the response variable, *β*_0_ is a constant and *β_i_*, *β_ii_*, and *β_ij_* are the coefficients for linearity, square and interaction, respectively. *X_i_* and *X_j_* are the levels of the independent variables. The model was built based on confidence levels of 95%.

### 3.5. Fatty Acid Composition

FAMEs of extracted oils were prepared using a fatty acid methylation method [28]. The FAMEs were identified by gas chromatography (GC) using an Agilent 6890N GC-FID (Agilent Technologies, Folsom, CA, USA) equipped with a fused silica capillary column (30 mm × 0.25 mm i.d). Thickness of the polyethylglycol-coated film was 0.32 μm. A solution of 1 μL of hexane containing the methylated sample was injected with a split ratio of 100:1. The inlet temperature was 220 °C and the detector temperature was 260 °C. The initial oven temperature was 170 °C and was then increased to 240 °C at a rate of 5 °C/min, and held at 240 °C for 5 min. Nitrogen was used as the carrier gas at a flow rate of 1.4 mL/min. The FAMEs were identified and quantified by comparison with standard FAME mix standards.

### 3.6. Antioxidant Activity

The antioxidant activity of tea seed oils extracted using both methods was determined using a DPPH assay, based on the method proposed by Brand-Williams *et al*. [29]. A 2 mL sample of the extracted oil at various concentrations (10–160 mg/mL) in DMSO was added to 2 mL of 0.005% (w/v) ethanolic DPPH solution. The decrease in absorbance of DPPH at 517 nm was measured by a UV/Vis spectrophotometer (HP8453, Hewlett Packard, USA) after incubation for 30 min at 30 °C in the dark. The radical-scavenging ability of the tested samples was calculated according to the following formula:

$$\text{Scavenging DPPH}\;(\%)=[(A_{\text{cont}}-A_{\text{sample}})/A_{\text{cont}}]\times 100$$

where *A*_cont_ and *A*_sample_ were defined as absorbance of the control and extracted oils, respectively.

### 3.7. Statistical Analysis

The results were statistically evaluated by analysis of variance (ANOVA) followed by a Tukey’s range test. Statistical tests were carried out using the SAS system for Windows (Version 9.1, SAS Institute Inc., Cary, NC, USA), and *p* < 0.05 was regarded as statistically significant.

## 4. Conclusions

Based on the results obtained, we conclude RSM is effective for determining the optimal conditions for tea seed oil extraction by SC-CO_2_. This type of extraction, which does not require modifiers, is a promising alternative for efficient extraction of edible oil from tea seed although further work is required to refine various parameters and to ensure quality is optimal in industrial processing. Moreover, tea seed oil extracted by SC-CO_2_ is highly edible and has good antioxidant activity, and therefore may play a potential role as a health-promoting food resource in human diets.

## Figures and Tables

**Table 1:** Results of the central composite design for the extraction of tea seed oil.

Runs	Coded variables			Oil yield (%)	

	*X*_1_ (°C)	*X*_2_ (min)	*X*_3_ (MPa)	Exp. *	Pred. **
1	−1	−1	−1	16.9	16.4
2	1	−1	−1	14.6	14.8
3	−1	1	−1	26.4	26.2
4	1	1	−1	27.1	27.0
5	−1	−1	1	26.1	25.8
6	1	−1	1	25.2	25.0
7	−1	1	1	28.0	27.4
8	1	1	1	28.8	29.0
9	−1.68	0	0	25.8	26.6
10	1.68	0	0	26.7	26.5
11	0	−1.68	0	16.4	16.7
12	0	1.68	0	28.1	28.3
13	0	0	−1.68	18.3	18.5
14	0	0	1.68	27.8	28.1
15	0	0	0	26.6	27.2
16	0	0	0	26.5	27.2
17	0	0	0	27.9	27.2
18	0	0	0	26.6	27.2
19	0	0	0	27.8	27.2
20	0	0	0	28.1	27.2

* Exp. is expressed as experimental value;

** Pred. is expressed as predicted value.

**Table 2:** ANOVA table of variates.

Source	Sum of squares	df	Mean square	*F*-value	*p*-value
Model	373.26	9	41.47	89.63	<0.0001
*X*_1_	2.54 × 10^−3^	1	2.54 × 10^−3^	5.50E-03	0.9424
*X*_2_	162.97	1	162.97	352.19	<0.0001
*X*_3_	111.81	1	111.81	241.64	<0.0001
*X*_1_*X*_2_	2.76	1	2.76	5.97	0.0347
*X*_1_*X*_3_	0.28	1	0.28	0.61	0.4537
*X*_2_*X*_3_	34.03	1	34.03	73.54	<0.0001
*X*_1_^2^	0.90	1	0.90	1.94	0.1942
*X*_2_^2^	39.88	1	39.88	86.19	<0.0001
*X*_3_^2^	27.47	1	27.47	59.37	<0.0001
Residual	4.63	10	0.46		
Lack of fit	1.77	5	0.35	0.62	0.6932
Pure error	2.86	5	0.57		
Corrected total	377.89	19			

**Table 3:** Comparison of main fatty acid contents of tea seed oil by different methods.

Methods	C7:0 *	C16:0 *	C18:0 *	C18:1 *	C18:2 *	C20:1 *	SFA *	MUFA *	PUFA *	Others
SC-CO_2_	0.7	15.3	3.3	57.5	22.3	0.9	18.6	58.4	22.3	0.7
Soxhlet	0.7	17.7	3.8	52.9	24.2	0.7	21.5	53.6	24.2	0.7

* C7:0, heptanoic acid; C16:0, palmitic acid; C18:0, stearic acid; C18:1, oleic acid; C18:2, linoleic acid; C20:1, gondoic acid; SAF, saturated fatty acids; MUFA, monounsaturated fatty acids; PUFA, polyunsaturated fatty acids.

**Table 4:** Code levels of independent variables used in the RSM design.

Independent variables	Coded symbols	Levels				

		−1.68	−1	0	1	1.68
Extraction temperature (°C)	*X*_1_	31.59	35	40	45	48.41
Extraction time (min)	*X*_2_	36.36	50	70	90	103.64
Extraction pressure (Mpa)	*X*_3_	21.59	25	30	35	38.41

## References

[1] Khan N., Mukhtar H. (2007). Tea polyphenols for health promotion. Life Sci.

[2] Tian H.Z., Qiu A.Y., Shi X.H. (2004). Extraction of tea seed polysaccharide. China Oil (Chinese).

[3] Ravichandran R., Dhandapani M. (1992). Composition characteristics and potential uses of south Indian tea seeds. J. Food Sci. Technol.

[4] Sahari M.A., Ataii D., Hamedi M. (2004). Characteristics of tea seed oil in comparison with sunflower and olive oils and its effect as a natural antioxidant. Am. Oil Chem. Soc.

[5] Sprecher H. (1981). Biochemistry of essential fatty acids. Prog. Lipid Res.

[6] Bourre J.M. (2006). Effects of nutrients (in food) on the structure and function of the nervous system: Update on dietary requirements for brain. Part 1: Micronutrients. J. Nutr. Health Aging.

[7] Kim N.H., Choi S.K., Kim S.J., Moon P.D., Lim H.S., Choi I.Y., Na H.J., An H.J., Myung N.Y., Jeong H.J. (2008). Green tea seed oil reduces weight gain in C57BL/6J mice and influences adipocyte differentiation by suppressing peroxisome proliferator-activated receptor-γ. Pflugers Arch.: Eur. J. Physiol.

[8] Rajaei A., Barzegar M., Yamini Y. (2005). Supercritical fluid extraction of tea seed oil and its comparison with solvent extraction. Eur. Food Res. Technol.

[9] Machmudah S., Kawahito Y., Sasaki M., Goto M. (2007). Supercritical CO_2_ extraction of rosehip seed oil: Fatty acids composition and process optimization. J. Supercrit. Fluids.

[10] Jiao S., Li D., Huang Z., Zhang Z., Bhandari B., Chen X.D., Mao Z (2008). Optimization of supercritical carbon dioxide extraction of flaxseed oil using response surface methodology. Int. J. Food Eng.

[11] Boutin O., Badens E. (2009). Extraction from oleaginous seeds using supercritical CO_2_: Experimental design and products quality. J. Food Eng.

[12] Mitra P., Ramaswamy H.S., Chang K.S. (2009). Pumpkin (*Cucurbita maxima*) seed oil extraction using supercritical carbon dioxide and physicochemical properties of the oil. J. Food Eng.

[13] Ixtaina V.Y., Vega A., Nolasco S.M., Tomás M.C., Gimeno M., Bárzana E., Tecante A. (2010). Supercritical carbon dioxide extraction of oil from Mexican chia seed (*Salvia hispanica* L.): Characterization and process optimization. J. Supercrit. Fluids.

[14] Ballard T.S., Mallikarjunan P., Zhou K., O’Keefe S.F. (2009). Optimizing the extraction of phenolic antioxidants from peanut skins using response surface methodology. J. Agric. Food Chem.

[15] Bhattacharjee P., Singhal R.S., Tiwari S.R. (2007). Supercritical carbon dioxide extraction of cottonseed oil. J. Food Eng.

[16] Liu S., Yang F., Zhang C., Ji H., Hong P., Deng C. (2009). Optimization of process parameters for supercritical carbon dioxide extraction of *Passiflora* seed oil by response surface methodology. J. Supercrit. Fluids.

[17] Shao P., Sun P., Ying Y. (2008). Response surface optimization of wheat germ oil yield by supercritical carbon dioxide extraction. Food Bioprod. Process.

[18] Wei Z.J., Liao A.M., Zhang H.X., Liu J., Jiang S.T. (2009). Optimization of supercritical carbon dioxide extraction of silkworm pupal oil applying the response surface methodology. Bioresour. Technol.

[19] Celebi N., Yildiz N., Demir A.S., Calimli A. (2008). Optimization of benzoin synthesis in supercritical carbon dioxide by response surface methodology (RSM). J. Supercrit. Fluids.

[20] Wang L., Yang B., Du X., Yi C. (2008). Optimisation of supercritical fluid extraction of flavonoids from *Pueraria lobata*. Food Chem.

[21] Liu J., Lin S., Wang Z., Wang C., Wang E., Zhang Y. (2011). Supercritical fluid extraction of flavonoids from *Maydis stigma* and its nitrite-scavenging ability. Food Bioprod. Process.

[22] Thana P., Machmudah S., Goto M., Sasaki M., Pavasant P., Shotipruk A. (2008). Response surface methodology to supercritical carbon dioxide extraction of astaxanthin from *Haematococcus pluvialis*. Bioresource Technol.

[23] Tokue C., Kataoka E., Tanimura W. (1989). Characterization of lipids in tea (*Camellia sinensis*) seeds cultivated in Taiwan and Japan. J. Jpn. Soc. Nutr. Food Sci. (Jpn.).

[24] Ravichandran R. (1993). Fat stability and amino acids in south Indian tea seeds. Int. J. Food Sci. Technol.

[25] Kris-Etherton P.M. (1999). Monounsaturated fatty acids and risk of cardiovascular disease. Circulation.

[26] Rajaei A., Barzegar M., Sahar I.M. (2008). Comparison of antioxidative effect of tea and sesame seed oils extracted by different methods. J. Agric. Sci.

[27] Li B., Xu Y., Jin Y.X., Wu Y.Y., Tu Y.Y. (2010). Response surface optimization of supercritical fluid extraction of kaempferol glycosides from tea seed cake. Ind. Crop. Prod.

[28] Wenli Y., Yaping Z., Jingjing C., Bo S. (2004). Comparison of two kinds of pumpkin seed oils obtained by supercritical CO_2_ extraction. Eur. J. Lipid Sci. Technol.

[29] Brand-Williams W., Cuvelier M., Berset C. (1995). Use of a free radical method to evaluate antioxidant activity. LWT-Food Sci. Technol.

